# Molecular characterization of SUT Gene Family in Solanaceae with emphasis on expression analysis of pepper genes during development and stresses

**DOI:** 10.1080/21655979.2022.2107701

**Published:** 2022-10-19

**Authors:** Wenqi Chen, Weiping Diao, Huiqing Liu, Qinwei Guo, Qiuping Song, Guangjun Guo, Hongjian Wan, Yougen Chen

**Affiliations:** a College of Horticulture, Anhui Agricultural University, Hefei, China; b State Key Laboratory for Managing Biotic and Chemical Threats to the Quality and Safety of Agro-Products, Institute of Vegetables, Zhejiang Academy of Agricultural Sciences, Hangzhou 310021, PR China; c Institute of Vegetable crops, Jiangsu Academy of Agricultural Sciences, Jiangsu Key Laboratory for Horticultural Crop Genetic Improvement, Nanjing, 210014, China; d Quzhou Academy of Agricultural and Forestry Sciences, Quzhou, 324000, China

**Keywords:** Solanaceae, pepper, sucrose transporter, gene family, evolution relationship

## Abstract

Sucrose, an essential carbohydrate, is transported from source to sink organs in the phloem and is involved in a variety of physiological and metabolic processes in plants. Sucrose transporter proteins (SUTs) may play significant parts in the phloem loading and unloading of sucrose. In our study, the *SUT* gene family was identified in four Solanaceae species (*Capsicum annuum, Solanum lycopersicum, S. melongena*, and *S. tuberosum*) and other 14 plant species ranged from lower and high plants. The comprehensive analysis was performed by integration of chromosomal distribution, gene structure, conserved motifs, evolutionary relationship and expression profiles during pepper growth under stresses. Chromosome mapping revealed that *SUT* genes in Solanaceae were distributed on chromosomes 4, 10 and 11. Gene structure analysis showed that the subgroup 1 members have the same number of introns and exons. All the *SUTs* had 12 transmembrane structural domains exception from *CaSUT2* and *SmSUT2*, indicating that a structure variation might occurred among the Solanaceae SUT proteins. We also found a total of 20 conserved motifs, with over half of them shared by all SUT proteins, and the SUT proteins from the same subgroup shared common motifs. Phylogenetic analysis divided a total of 72 *SUT* genes in the plant species tested into three groups, and subgroup 1 might have diverged from a single common ancestor prior to the mono-dicot split. Finally, expression levels of *CaSUTs* were induced significantly under heat, cold, and salt treatments, indicating diverse functions of the *CaSUTs* to adapt to adverse environments.

## Graphical Abstract

**Figure:**
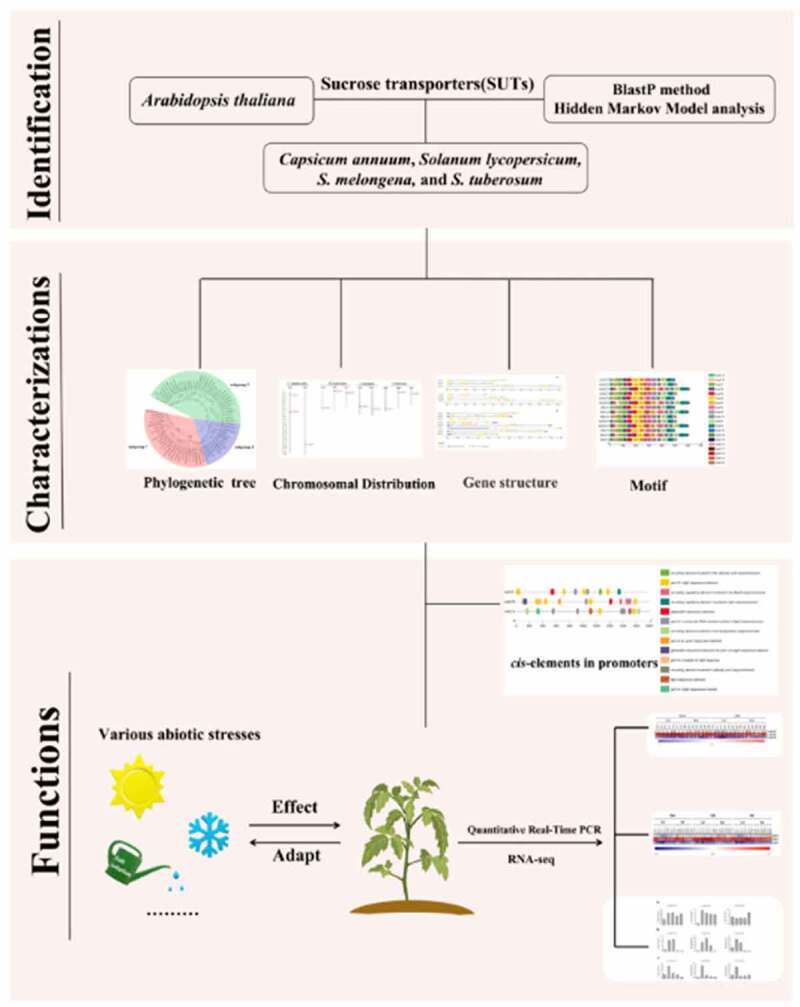

## 1. Introduction

Sucrose, as the principal carbohydrate, is the final product of photosynthesis in most plants, and it is closely related to various physiological and metabolic actions [1,2]. Moreover, the long-distance transportation of sucrose from ‘source’ to ‘sink’ tissues is completed in the phloem, and there are two pathways for sucrose transport into the functioning phloem sieve elements (SEs): symplasmic pathway and apoplasmic pathway. Among them, the symplasmic transportation process does not rely on transmembrane transporters. Nevertheless, for the apoplasmic pathway process, the loading and unloading processes of sucrose are accomplished depending on the sucrose transporters (SUTs) [3–7].

The *SUT* genes are members of the ancient major facilitator superfamily (MFS) which can be traced back to prokaryotes over 3.5 billion years ago [8,9,10]. In addition, the SUT proteins contain a total of 12 hydrophobic transmembrane structural domains in the form of α-helices, which is one of the common structural characteristics for MFS members. Moreover, previous studies also reported that a hydrophilic loop structure exists between transmembrane region 6 and 7 [4,11–17].

As early as 1992, Riesmeier [18] had isolated and characterized the first *SUT* gene from spinach (*Spinacea oleracea* L.). Subsequently, more *SUT* genes were identified in various plants. In 2010, a phylogenetic tree of *SUT* genes from multiple plant species was reported [15], and the result showed that *SUT*s can be divided into five groups (SUT1-SUT5). Among them, the SUT1 is unique to dicotyledons, and the two groups (SUT3 and SUT5) are monocot-specific, whereas SUT2 and SUT4 contain members from dicotyledons and monocotyledons [15]. Furthermore, members of different SUT groups perform diversified physiological functions [19]. The SUT1 members have a high affinity for sucrose and play an essential role in the loading and unloading of sucrose in the phloem [15,20,21]. On the contrary, members of the SUT2 and SUT4 show low affinity, but surprisingly, contribute to the rapid transport of sucrose at high sucrose concentrations [12,22,23]. Moreover, monocots employ members from the SUT3 and SUT5 groups to accomplish the sucrose loading process in phloem [24].

The *SUT*s, as sucrose transporters, play vital roles during the growth and development of plants [25–28]. In cacao (*Theobroma cacao* L.) and peas (*Pisum sativum* L.), *TcSUT* and *PsSUT1* genes are highly expressed in seeds and leaves, respectively, leading to higher seed yields [29,30]. Furthermore, in rice (*Oryza sativa* L.), down-regulated expression of *OsSUT1* in source and sink tissues decreased the yield and quality [31]. Additionally, researchers also found that overexpression of *PbSUT2* in transgenic tomato plants resulted in early flowering [32], and *AtSUC9* mutant exhibit delayed flowering [33]. The *SUT*s also play key roles in anthocyanin biosynthesis and senescence in grape [34,35]. In addition, *DcSUT2* is expressed in the storage parenchyma tissues of carrot (*Daucus carota* L.) taproots, which can transport sucrose into the taproot for storage [36].

On the other hand, researchers reported that *SUT* genes were indispensable for maintaining plant normal growth and development under various stresses. In rice, the increased expression level of *OsSUT1* contributed to maintaining the seed quality under high-temperature stress [37]. Both abscisic acid (ABA) and gibberellin acid (GA) induced expression of *MdSUT1*, which improves the abiotic tolerance of apple [9,21]. Furthermore, expression levels of *AgSUT1* were reduced in all organs of celery (*Apium graveolens* L.) under salt stress, which allowed for maintaining common physiological function [38].

Solanaceae is one of the most important botany families, containing many model plants such as tomato (*Solanum lycopersicum*) and tobacco (*Nicotiana tabacum* L). As a vital economic crop, pepper (*Capsicum annum* L.), a member of the Solanaceae family, is native to the tropical regions of Latin America, and cultivated widely in the world. Although the *SUT* gene family had been identified in the whole genome in a variety of plants [23–30], little was reported in pepper.

Sucrose transporters (SUTs) may play essential roles in assimilating sucrose allocation within cells and at the whole-plant level, and the expression levels of *SUT*s are affected by various abiotic stresses. Therefore, this study performed genome-wide identification and characterization of the members of the *SUT* gene families in Solanaceae species. In addition, we conducted Quantitative Real-Time PCR to analyze the expression patterns of the *SUT*s in pepper under different abiotic stresses. The outcome of this study provides valuable insights into the evolution of *SUT* genes in Solanaceae, and further understanding of the biological function of the *SUTs* throughout growth and development.

## 2. Materials and Methods

### 2.1. *Database Retrieval and Genome-wide Identification of* SUT *Genes in Pepper and Sixteen Other Plant Species*

The whole-genome reference sequences of *Capsicum annuum* L. were downloaded from the pepper genome database (http://passport.pepper.snu.ac.kr/?t=PGENOME and https://solgenomics.net) . The genome information about *Arabidopsis thaliana, Solanum lycopersicum, Oryza sativa, Vitis vinifera, Solanum melongena, Sorghum bicolor, Citrullus lanatus, Cucurbita moschata, Beta vulgaris, Cucumis sativus, Phaseolus vulgaris, Theobroma cacao, Zea mays, Solanum tuberosum, Daucus carota and Lagenaria siceraria* was obtained from Phytozome v13 (https://phytozome-next.jgi.doe.gov/blast-search), Sol Genomics Network (https://solgenomics.net), Eggplant Genome Database (http://eggplant-hq.cn/Eggplant/home/index) and Potato Genomics Resource (http://spuddb.uga.edu/index.shtml). Then, the local database had set up. The *SUT*s belong to the MFS-2 family, and the Hidden Markov Model (HMM) [39]file corresponding to the MFS-2 domain (PF13347.6) was retrieved from Pfam 34.0 (http://pfam.xfam.org), which was employed to identify putative SUT proteins. Next, we carried out a Blastp [40] search using online databases with a set E-value threshold of 1^e−5^. All predicted candidates with the SUT domain were selected for the subsequent analysis.

### 2.2. Characterization of SUT Proteins in Solanaceae and Phylogenetic Analysis

The ExPasy (https://web.expasy.org/compute_pi/) was utilized for analyzing the characteristics of the SUT protein, including molecular weight (Mw) and protein isoelectric point(pI) [41]. Prediction of transmembrane (TM) helix domains of SUT proteins was performed using TMHMM Server v2.0 (http://www.cbs.dtu.dk/services/TMHMM/). To determine the conserved motifs of SUT proteins in Solanaceae, we had chosen the online program MEME (https://meme-suite.org/meme/tools/meme) with the default parameter with the exception that the maximum number was set to 20. We used TBtools [42] to visualize the conserved motifs in SUT proteins by converting the motif location. To examine the phylogenetic relationships of *SUT* genes, representative *SUT* genes from *Arabidopsis thaliana, Solanum lycopersicum, Oryza sativa, Vitis vinifera, Solanum melongena, Sorghum bicolor, Citrullus lanatus, Cucurbita moschata, Beta vulgaris, Cucumis sativus, Phaseolus vulgaris, Theobroma cacao, Zea mays, Solanum tuberosum, Daucus carota and Lagenaria siceraria*, as well as *SUT* genes from *Capsicum annuum*, were used to build a phylogenetic tree. Multiple sequence alignment was executed using ClustalW [43] with default settings. The un-rooted phylogenetic tree was produced by MEGAX with a bootstrap of 1000 replicates, and the neighbor-joining (NJ) method was used [44].

### 2.3. *Gene Structure and Chromosomal Localization of* SUT *Genes in Solanaceae*

The Gene Structure Display Server (GSDS 2.0, http://gsds.gao-lab.org) [45] was applied to analyze the gene structure of all *SUTs* identified in *C. annuum, S. lycopersicum, S. melongena* and *S. tuberosum*. The software MapChart was used to visualize the physical location information [46].

### 2.4. *Analysis of Promoter Regions of* CaSUT *Gene Family Members*

The 2000 bp DNA sequence (promoter sequences) of the upstream region of each *CaSUT* gene was gathered from the Sol Genomics Network database (https://solgenomics.net). The online tool PlantCARE (http://bioinformatics.psb.ugent.be/webtools/plantcare/html/) [47] was used to predict *cis*-acting elements in promoter regions of *CaSUT* family genes. The *cis*-acting structure of each member of *CaSUT* family was visualized using the software TBtools [42].

### 2.5. *Expression Analysis of* CaSUT *Genes*

To analyze the expression patterns of *CaSUT* genes in different organs, the Pepper Functional Genomics Database (PFGD; https://www.pfgd.org) was applied. The plants were cultivated in a natural glasshouse with daily temperatures ranging from 25°C to 29°C and night temperatures ranging from 16°C to 20°C. Seven tissues were selected, including leaf, flower, petal, ovary, stamens, fruit and seed. Flowers at the fourth bifurcation were tagged on the day of opening to obtain fruit samples at various developmental stages. Fruits were gathered 3, 7, 10, 15, 20, 25, 30, 35, 40, 45 and 50 days after flowering (DAF) from the tagged flowers. Floral buds were collected at 9 different stages based on their size. The fully blossomed flowers were divided into petals, ovary with stigma, and stamen. Leaf samples were collected at 2, 5, 10, 15, 20, 25, 30, 40, and 50 days after emergence (DAE).

### 2.6. *Expression Profiles of* CaSUT *Genes using RNA-seq*

To investigate the expression profiles of predicted *CaSUT* genes in response to different stresses such as heat, cold, methyl jasmonic acid (MeJA), abscisic acid (ABA) and salt, the complete RNA-seq data for various environmental stresses were downloaded from the database PFGD (https://www.pfgd.org). Seedlings of line 6421 were treated with 10 μM MeJA and 30 μM ABA. These two hormones (MeJA and ABA) were chosen and added to the nutrient solution. The seedlings were moved into a growth chamber at 42°C (heat treatment) or 10°C (cold stress treatment), and salt stress was given by adding NaCl to a final concentration of 200 mM in the nutrient solution. Plants in the control group were just given nutrient solutions. All stress treatments had been performed to seedlings of pepper which was 40 days old, and the stresses treatment time set to 0, 0.5, 1, 3, 6, 12 and 24 hours with each five biological replicas. Further, the treated plants and control plants were collected respectively. Then, the raw Reads Per Kilo bases per Million mapped Reads (RPKM) values for candidate genes were normalized. Finally, heat maps of *CaSUT* gene expression pattern in each tissue and under stress treatments were generated using Multi Experiment Viewer (MeV) [48].

### 2.7. Plant growth and stress treatment

*C. annuum* cultivar D50 provided by the Zhejiang Academy of Agricultural Sciences was used to perform the expression of candidate genes. Plants were grown at room temperature of 25 ± 1°C, 16 h light/20 ± 1°C, 8 h night. Plants that were 6 weeks old and had similar growth conditions were chosen for cold, heat, and salt treatments. For cold stress, seedlings were transferred to a growth chamber at 4°C, and leaves were collected at 1 h, 3 h, 6 h, and 12 h. For heat stress, seedlings were maintained at 42°C for 0.5 h, 1 h, 4.5 h, and 6 h, then leaves were collected respectively. A final concentration of 400 mM NaCl was used for salt stress and leaves were collected at 1 h, 6 h, 12 h, and 24 h. No less than 3 plants were selected for each abiotic stress treatment. All samples were quick-frozen in liquid nitrogen and stored at −80°C for RNA isolation.

### 2.8. RNA Extraction and Quantitative Real-Time PCR Analysis

Total RNA was isolated from pepper leaves using the E.Z.N.A.R® Plant RNA Kit (OMEGA, United States). To avoid degradation, all steps were accomplished at low temperature. The first strand cDNA was synthesized from 1 µg of total RNA using FastKing RT Kit (with gDNase) (TIANGEN, China) according to the manufacturer’s instructions.

Gene-specific primers for Quantitative Real-Time PCR (qRT-PCR) were designed using the Genscript online tool (https://www.genscript.com/tools/real-time-pcr-taqman-primer-design-tool). The pepper GAPDH gene was utilized as an endogenous reference gene for normalizing the expression levels. The reaction system of qRT-PCR analysis followed the instructions of SYBR Green Master Mix reagent of Vazyme with 20 µl reaction mixture of volume on CFX96 Real-Time System (Bio-Rad, United States) . All qRT-PCRs were performed in triplicate and the expression data were calculated by the 2^−ΔΔCt^ method.

## 3. Results

### 3.1. *Identification of* SUT *Gene Family*

The *SUT*s have been identified in different plant species, such as *Arabidopsis* [49]. Nevertheless, systematic analysis of the *SUT* gene family in peppers with other Solanaceae had not been reported. Here, we identified a total of 14 *SUT* genes in *C. annuum* cv zunla, *C. annuum* cv CM334, *S. lycopersicum, S. melongena* and *S. tuberosum*, using the Hidden Markov Model (HMM) profile (PF13347.6) and Blastp analysis. The *SUT* genes in *C. annuum* zunla, *C. annuum* CM334, *S. lycopersicum, S. melongena*, and *S. tuberosum* were assigned as ‘*CaSUT*’, ‘*CASUT*’, ‘*SlSUT*’, ‘*SmSUT*’ and ‘*StSUT*’, respectively (Table 1). In addition, the members of the *SUT* gene family from 13 other plant species were selected for illustrating their evolutionary relationships, including *Arabidopsis*, rice, grape, sorghum, watermelon, pumpkin, beet, cucumber, bean, cocoa, corn, carrot, and bottle gourd (Table S1). Compared to the number of *SUT* genes in Solanaceae, the number of pepper *SUT* genes is similar to tomato and potato, which is higher than in eggplant. Moreover, the number of *SUT* genes range from 2 to 3 in the Solanaceae species tested, which were less than the numbers in Poaceae (4–5), *Arabidopsis* (9), and Cucurbitaceae (3–4). Further analysis showed that *SUT* genes in Solanaceae species encode 497 to 611 amino acids (aa). The predicted molecular weight (Mw) of the *SUTs* varied from 54.27 KDa (*StSUT1*) to 65.56 KDa (*CaSUT3* and *CASUT3*). The theoretical isoelectric point (pI) value ranges from 6 to 9.32. Among them, *CaSUT3* (6), *CASUT3* (6), *SlSUT2* (5.95), *SmSUT2* (6.46), and *StSUT2* (6.02) proteins were weakly acidic with pI values less than 7, the remaining pI values were above 7, which were typical alkaline proteins. In contrast to *SUT* genes in Solanaceae, the pI values of *SUT* genes of beet and grape were both greater than 7, indicating that their SUT proteins were both basic. It has been inferred that function of the SUT genes could be diversified in different species.

**Table 1.** Characteristics of SUT genes in Solanaceae species.

Species	Gene ID	name	subgroup	ORF Length (bp)	Protein Length(aa)	Isoelectric point	Molecular weight(kDa)	Number of predicted TMHs
*Capsicum annuum* zunla	Capana04g000792	CaSUT1	subgroup 2	1506	501	9	54.28	12
	Capana04g001621	CaSUT2	subgroup 3	1551	516	9.32	54.76	12
	Capana11g001648	CaSUT3	subgroup 1	1836	611	6	65.56	12
*Capsicum annuum CM334*	CA04g11150	CASUT1	-	1551	516	9.32	54.76	12
	CA04g17270	CASUT2	-	1494	497	8.88	53.81	10
	CA11g05110	CASUT3	-	1836	611	6	65.56	12
*Solanum lycopersicum*	Solyc04g076960	SlSUT1	subgroup 2	1503	500	8.76	54.24	12
	Solyc05g007190	SlSUT2	subgroup 1	1815	604	5.95	65	12
	Solyc11g017010	SlSUT3	subgroup 3	1536	511	9.31	54.15	12
*Solanum melongena*	Smechr0401253	SmSUT1	subgroup 3	1548	515	9.29	54.65	12
	Smechr1001581	SmSUT2	subgroup 1	1812	603	6.46	64.84	11
*Solanum tuberosum*	Soltu.DM.04G031670	StSUT1	subgroup 2	1503	500	9.03	54.27	12
	Soltu.DM.05G006180	StSUT2	subgroup 1	1812	603	6.02	64.85	12
	Soltu.DM.11G010180	StSUT3	subgroup 3	1548	515	9.18	54.64	12

Note: ORF, Opening Reading Frame; aa, amino acid

### 3.2. *Phylogenetic Analysis of* SUT *Genes in Major Plant Species*

**Figure 1.**
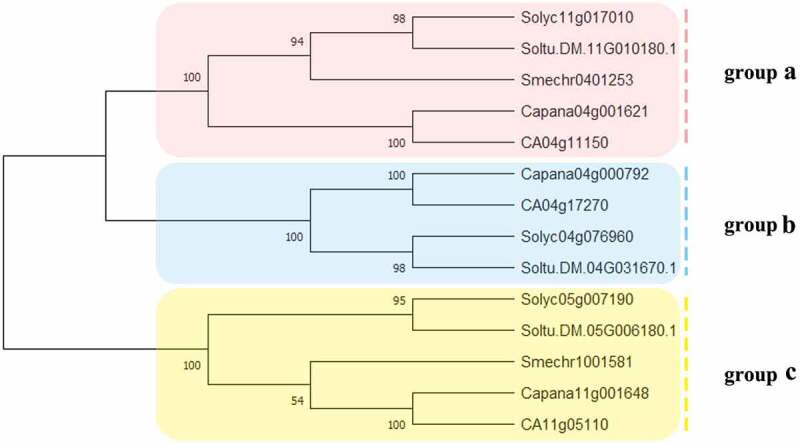
The phylogenetic analysis of SUT proteins from *C. annuum, S. lycopersicum, S.melongena, S. tuberosum*. Fourteen SUT proteins were used to construct the NJ tree with 1000 bootstraps based on the protein sequences. The SUT proteins were grouped into 3 groups.

To study the evolutionary relationships among the Solanaceae SUT proteins, SUT proteins in 4 Solanaceae species (*C. annuum; S. lycopersicum; S. melongena; S. tuberosum)* were identified and used in phylogenetic analysis (Figure 1). Solanaceae SUT proteins were divided into 3 groups, and group B did not contain eggplant.

**Figure 2.**
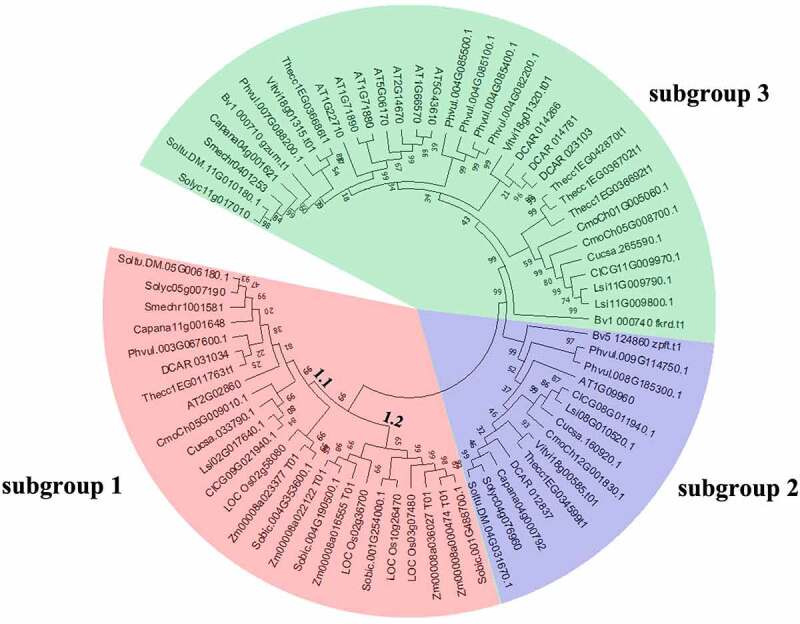
Phylogenetic relationships among *SUT* genes in 17 plant species. The species’ abbreviations were as follows: *C. annuum* (Ca), *A. thaliana*. (At), *S. lycopersicum* (Sl), *O. sativa* (Os), *V. vinifera* (Vv), *S. melongena* (Sm), *S. bicolor* (Sb), *C. lanatus* (Cl), *C. moschata* (Cm), *B. vulgaris* (Bv), *C. sativus* (Cs), *P. vulgaris* (Pv), *T. cacao* (Tc), *Z. mays* (Zm), *S. tuberosum* (St), *D. carota* (Dc) and *L.siceraria* (Ls). The phylogenetic tree was constructed using the neighbor-joining (NJ) method and with 1000 bootstrap replications. The numbers at the nodes represented bootstrap percentage values. 72 SUT proteins were divided into 3 subgroups. Three subgroups were marked with different background colors.

To assess the phylogenetic relationship among pepper SUT proteins with other 16 plant species, we constructed an unrooted phylogenetic tree (Figure 2) with a total of 72 *SUT* genes from *C. annuum, A. thaliana, S. lycopersicum, O. sativa, V. vinifera, S. melongena, S. bicolor, C. lanatus, C. moschata, B. vulgaris, C. sativus, P. vulgaris, T. cacao, Z. mays, S. tuberosum, D. carota and L.siceraria*. The result exhibited that the 72 *SUT* genes were also divided into 3 clades (designated as subgroups 1 to 3), including 25, 14, and 33 members, respectively. Among them, subgroup 1 was divided into 2 parts; subgroup 1.1 composed of 15 *SUT* genes from both monocot and dicot species. Subgroup 1.2 was a monocot-specific subgroup containing 3 *OsSUTs*, 4 *ZmSUTs*, and 3 *SbSUTs*, which supported earlier research [15]. The members of subgroup 1 were from the other 16 plant species tested except for *B. vulgaris*. Subgroup 2 contained at least one member from *A. thaliana, S. tuberosum, S. lycopersicum, C. annuum, D. carota, T. cacao, V. vinifera, C. moschata, C. sativus, L.siceraria, C. lanatus, P. vulgaris* and *B. vulgaris*. Subgroup 3 was the largest group and contained *SUTs* exclusively from dicot species. Moreover, more *AtSUTs* were placed in subgroup 3 than in other two subgroups. Interestingly, all the members in subgroup 2 and subgroup 3 were from dicotyledons, corresponding well to the SUT1 clade [50], and demonstrating the close evolutionary link between homologous SUTs. Notably, same as the most Solanaceae SUTs, pepper *SUTs* were found in 3 subgroups.

### 3.3. *Chromosomal Distribution of* SUT *Genes*

**Figure 3.**
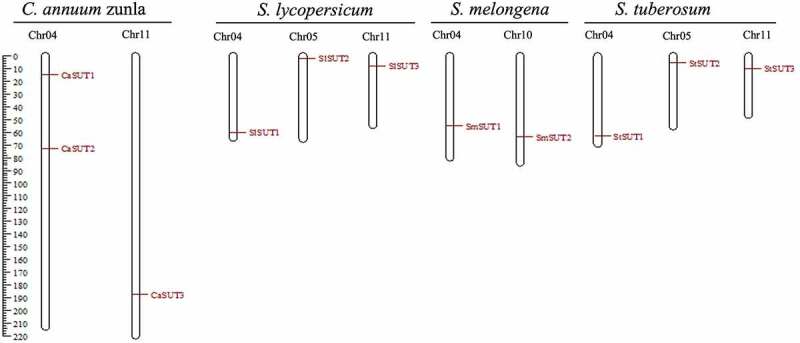
Distribution of *CaSUT* genes and Solanaceae *SUTs* gene on the chromosomes. The *SUTs* positions were shown as red lines. Chromosome numbers are shown at the top of each bar.

To understand the distribution of *CaSUT* genes and Solanaceae *SUTs* gene on the chromosomes, we constructed a chromosomal localization map of members of the *SUT* genes family (Figure 3). The result showed that the 3 *CaSUT* genes were mapped on 2 of 12 pepper chromosomes (chromosomes 4 and 11) with an uneven distribution pattern. Chromosome 4 contained 2 *CaSUT* genes, while chromosome 11 carried a single *CaSUT* gene. Meanwhile, we also found that most *SUTs* in Solanaceae except eggplant were located on chromosomes 4 and 11. Moreover, each of *SlSUT2* and *StSUT2* was located on chromosome 5.

### 3.4. *Intron-exon Structures and Conserved Motifs of* SUT *Genes*

**Figure 4.**
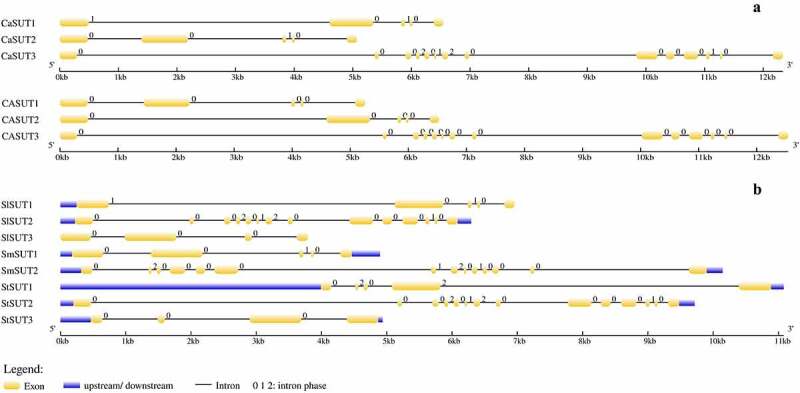
Genes structural organization of 14 *SUTs*. a) *C. annuum* Zunla and *C. annuum* cv CM334; b) *S. lycopersicum, S. melongena*, and *S. tuberosum*. Exons and introns were represented by yellow boxes and black lines, respectively. The. upstream/downstream were represented by blue boxes. The sizes of exons and introns are proportional to their sequence lengths. 0 = intron phase 0; 1 = intron phase 1; 2 = intron phase 2.

The structural diversity of multigene families means a direct signal of gene family expansion, and the conserved protein motifs play significant roles in the evolution of multiple gene families [51–53]. As described above (Table 1), *SUT* genes in Solanaceae had 10–12 TM helix domains, while each of the *CaSUTs* had 12 TM helix domains. The results supported the previous studies that sucrose transporters typically have 12 TM helix domains [11–16]. To visualize the structure of *SUT* genes, the introns and exons were characterized by comparing the cDNA sequences with the corresponding whole-genome DNA sequences (Figure 4). Structural analysis showed that *CaSUT3* contained the largest number of exons and introns (14 exons and 13 introns), and the remaining *CaSUT1* and *CaSUT2* contained 5 exons and 4 introns. Although from different pepper materials, the *SUT* genes in CM334 were identical in the number of introns and exons to *CaSUTs* (Figure 4 -a). The *CaSUT* genes which belonged to the same clade had similar intron-exon configurations. Although the lengths of *CaSUT1* and *CaSUT2* which both belong to the SUT1 clade [50] varied, similar intron-exon configurations and the same number of exons and introns were observed. The result indicated that closely related genes shared similar gene structures. Meanwhile, in each Solanaceae species (Figure 4 -b), there were unique *SUT* genes (*CaSUT3, SlSUT2, SmSUT2* and *StSUT2*) that differ significantly in length, introns and exons number (14 exons and 13 introns), when compared to other Solanaceae *SUTs* gene. Further analysis showed that all the above *SUT* genes from subgroup 1.

**Figure 5.**
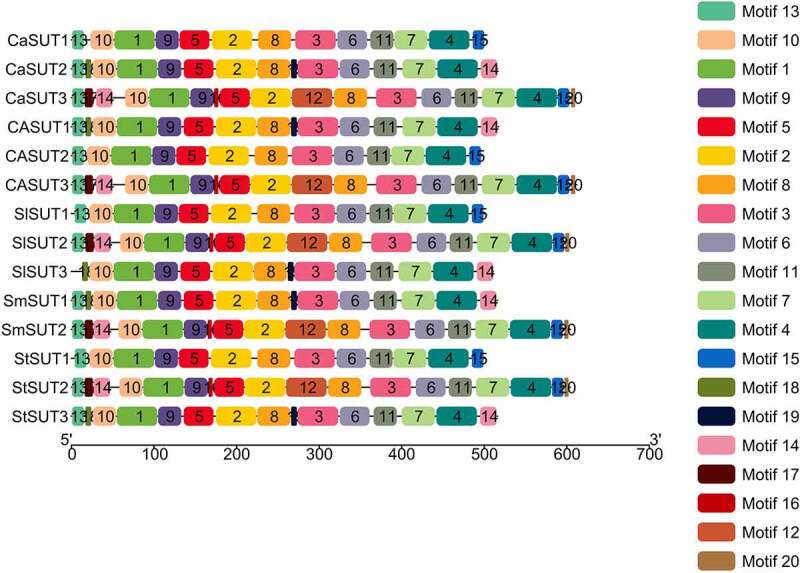
Distributions of conserved motifs in SUT proteins in Solanaceae species. Motifs of the SUT proteins identified by the online tool MEME. Different motifs are indicated by different colors and numbered 1–20. The same number in different proteins referred to the same motif.

Next, we predicted conserved motifs of SUT proteins in pepper and other Solanaceae plants using the MEME program, and found 20 conserved motifs (Motif 1 to Motif 20) (Figure 5). The length of these conserved motifs ranged from 6 to 50 amino acids. Each of the 14 *SUT* contained multiple motifs.

Among the 20 conserved motifs identified, 11 motifs (motif 1 − 11) were shared by all *SUTs* (Figure 5). In addition, motif 13 was identified in all of the SUT proteins except *SlSUT2. CaSUT3, SlSUT2, SmSUT2*, and *StSUT2* which all belonged to subgroup1 shared 4 common motifs (motif 12, 16, 17, and 20). While motif 14 and motif 15 were present in the most of *SUT* genes in Solanaceae, the 4 *SUTs* (*CaSUT3, SlSUT2, SmSUT2*, and *StSUT2*) which from subgroup 1 simultaneously shared the 2 motifs. The motif types and distribution of members of the same subgroups were highly conserved, suggesting a potential similar function. The motif 18 and motif 19 were only observed in members of subgroup 3(*CaSUT2, SlSUT3, SmSUT1*, and *StSUT3)*. Overall, the conserved motifs in different subfamilies varied, implying that the SUT proteins could have functional variation and evolutionary diversification.

### 3.5. *Promoter Analysis of Pepper* SUT *Genes*

**Figure 6.**
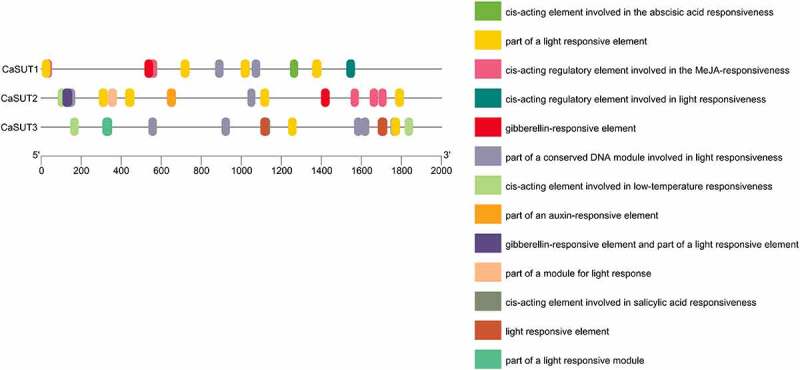
Promoters of *SUT* genes of pepper and their *cis-*elements analysis. The colored boxes represented different responsive *cis*-elements.

Several responsive *cis-*elements were found in promoter regions (2000bp) of pepper *SUT* genes using the online tool ‘PlantCare’ (Figure 6). These responsive *cis*-elements were mainly light, low-temperature, gibberellin, methyl jasmonic acid (MeJA), abscisic acid (ABA), salicylic acid (SA), and auxin responsive elements (Figure 6). As the result showed, light-responsive elements were present in promoter regions of all *CaSUT* family members. Except for *CaSUT3*, the remaining two *CaSUT* genes all had MeJA and gibberellin responsive elements (Figure 6). Auxin response elements were found in one of all the *CaSUTs* (*CaSUT2*) identified, whereas ABA and SA response elements were predicted in the promoter regions of *CaSUT1* and *CaSUT3*, respectively (Figure 6). The result suggested that *CaSUT* genes might be involved in multiple biological processes and had different regulatory pathways among different members during the growth and development processes of pepper.

### 3.6. *Expression Patterns of* CaSUT *Genes in Different Tissues*

**Figure 7.**
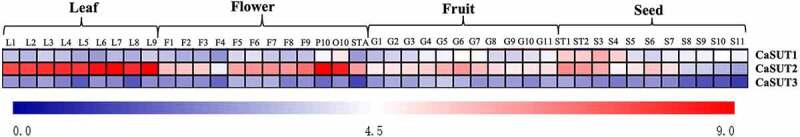
Heat map of the expression pattern of *SUT* genes in different tissues of pepper. L1-L9 indicated the expression of *CaSUT*s in leaves at 2, 5, 10, 15, 20, 25,30, 40 and 50 days after emergence; F1-F9 represented flower buds of different stages; P10: petals; O10: ovary; STA10: stamens; G1-G11 indicated the expression of *CaSUT*s in fruits at 3, 7, 10, 15, 20, 25, 30, 35, 40, 45 and 50 days after flowering, respectively; ST1 and ST2 represented seeds and placenta in fruits 10 and 15 days after flowering, respectively; S3-S11 indicated the expression of *CaSUT*s in leaves at 20, 25, 30, 35, 40, 45, 50, 55 and 60 days after flowering. Expression levels of the *CaSUT* genes were shown as the Log2 transformed FPKM values obtained from the RNA-Seq data. Heat maps were presented in blue/white/red colors that represent high/medium/low expression, respectively.

The transcript levels of *CaSUT* genes in 7 tissues/organs of pepper were investigated, including leaf, flower, petal, ovary, stamens, fruit and seed. All the *CaSUT* genes exhibited diverse expression patterns in different tissues/organs (Figure 7). Although similar gene structure and physicochemical characteristics of *CaSUT* genes were observed in the same subgroups, expression patterns of these genes in tissues/organs were different. As shown in Figure 7, *CaSUT3* was detected barely in all tested organs. On the contrary, *CaSUT1* and *CaSUT2* showed ubiquitous expression patterns in all investigated organs (Figure 7). Especially, these two genes showed the highest expression in the early stage of seeds development and leaves, respectively, suggesting their crucial function in the development of seeds and source organs (Figure 7). It was worth noting that *CaSUT2* was relatively highly expressed in all tested tissues in our study. In the present study, the result indicated that each *CaSUT* genes drive various function during the growth and development process of pepper.

### 3.7. *Expression of* CaSUT *Genes in Response to Salt, Cold, and High Temperature*

**Figure 8.**
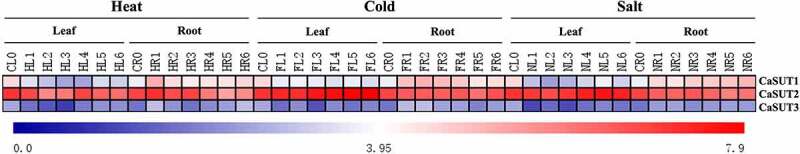
Heatmap showing the expression patterns of the *CaSUT* genes under different salt, cold and high temperature stress. CL0 indicated the expression of *CaSUT* in pepper leaves in the control group without any treatment; CR0 denotes the expression of *CaSUT* in pepper roots in the control group without any treatment; numbers 1–3 represented the expression of *CaSUT*s in leaves/roots of seeding at 0.5, 1, 3, 6, 12 and 24 hours after salt, cold and high temperature stress, respectively. HL/HR referred to the expression of *CaSUT* genes in leaves/roots under high temperature treatment; FL/FR indicated the expression of *CaSUT* genes in leaves/roots under cold temperature treatment; NL/NR indicated the expression of *CaSUT* genes in leaves/roots under salt stress. Heat maps were presented in blue/white/red colors that represent high/medium/low expression, respectively.

To further explore the responsive mechanism of the *CaSUT* gene family of members under abiotic stresses, we used RNA-seq to investigate the expression profiles of *CaSUT* genes under cold, heat, and salt treatment in roots and leaves of pepper. It should be noted that the expression of *CaSUT1* and *CaSUT3* were down-regulated in leaves, but in roots, their expressions were up-regulated (Figure 8). Under cold stress, the expression levels of *CaSUT1* and *CaSUT3* significantly increased in roots, but were down-regulated in leaves (Figure 8). Inversely, *CaSUT2* expression was up-regulated in leaves. Under salt stress, *CaSUT2* exhibited up-regulation in leaves, while the expression levels of the other two genes (*CaSUT1* and *CaSUT3*) decreased (Figure 8). In roots, *CaSUT1* and *CaSUT3* were up-regulated, and *CaSUT2* was suppressed (Figure 8).

### 3.8. *Expression of* CaSUT *Genes under Different Hormone Treatments*

**Figure 9.**
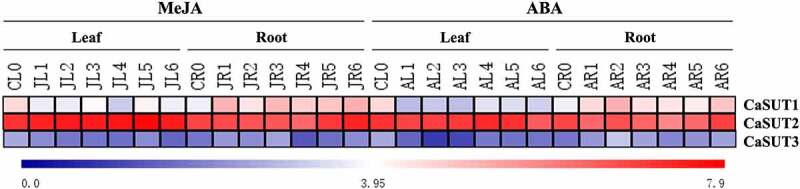
Heatmap showing the expression patterns of the *CaSUT* genes under methyl jasmonic acid (MeJA) and abscisic acid (ABA) treatment. CL0 indicated the expression of *CaSUT* in pepper leaves in the control group without any treatment; CR0 denoted the expression of *CaSUT* in pepper roots in the control group without any treatment; numbers 1–3 represented the expression of *CaSUT*s in leaves/roots of seeding at 0.5, 1, 3, 6, 12 and 24 hours after salt, cold and high temperature stress, respectively. JL/JR ped the expression of *CaSUT* genes in leaves/roots under MeJA treatment; AL/AR denoted the expression of *CaSUT* genes in leaves/roots under ABA treatment. Heat maps were presented in blue/white/red colors that represent high/medium/low expression, respectively.

The expression levels of *SUT*s were associated with various factors, such as the different kinds of phytohormones [54]. To better understand the function of *CaSUT* genes in response to exogenous phytohormones, we analyzed the expression pattern of pepper genes in response to MeJA and ABA in leaves and roots. Under MeJA treatment, we discovered that *CaSUT1, CaSUT2* and *CaSUT3* were up-regulated in roots (Figure 9). The transcript level of only one gene (*CaSUT2*) was slightly increased in leaves (Figure 9). Following ABA treatment, expression levels of *CaSUT1* and *CaSUT3* were down-regulated in leaves, while *CaSUT2* constantly showed a relatively high expression profile (Figure 9). Enhanced expression levels of two genes (*CaSUT1*and *CaSUT3*) in roots were observed after ABA treatment, and *CaSUT2* exhibited slight change in expression level (Figure 9).

### 3.9 *Response of* CaSUT *Genes to Various Abiotic Stress*

**Figure 10.**
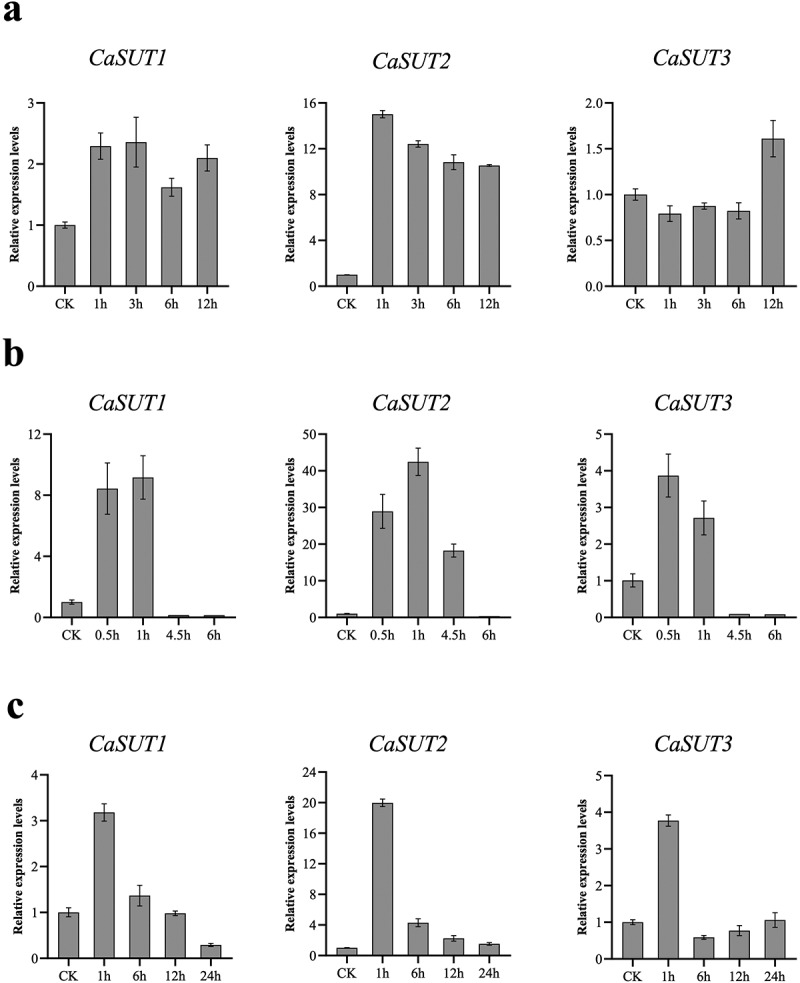
Expression patterns of *CaSUT* genes under abiotic stresses at different time points. CK referred to the untreated pepper seedlings that were only mock-treated with nutrient solution. The relative expression levels referred to the expression levels of the set time points after stresses were compared with those of untreated pepper seedlings. (a) cold stress, (b) heat stress, (c) salt stress.

To further investigate the protein roles of *SUT* genes in pepper responding to various stresses, pepper seedlings of D50 were subjected to cold, heat, and salt stresses. Then the qRT-PCR analysis of *CaSUT* was performed to measure the gene expression profiles in response to abiotic in leaves using 4 pairs of specific primers (Table 2). Under cold (4°C) stress, *CaSUT1* and *CaSUT2* genes were both induced in the whole stage of treatment, especially *CaSUT2*, whose expression was elevated more than ten-fold compared to that of the 0 h control (Figure 10 -a). In addition, the expression levels of *CaSUT1* reached its peak at 3 h, while *CaSUT2* peaked at 1 h. The transcription levels of *CaSUT3* were slightly inhibited in the early stage, while upregulated at 12 h after cold treatment (Figure 10 -a). Under heat (42°C) stress, *CaSUT2* showed significant up-regulation by 10- to 40-fold in leaves at 0.5 h, 1 h, and 4.5 h, and was inhibited at 6 h (Figure 10 -b). Meanwhile, the expression levels of *CaSUT1* and *CaSUT3* were initially upregulated, then repressed at 4.5 h and 6 h after heat treatment, respectively. Notably, the expression levels of *CaSUTs* exhibited differences across the four time points. *CaSUT1* and *CaSUT2* reached the maximum value at 1 h and *CaSUT3* reached a peak at 0.5 h (Figure 10 -b).

**Table 2.** The primers used in quantitative real-time PCR (qRT-PCR) analysis in this study.

Gene name	Forward primers	Reverse primers
GAPDH	ATGATGATGTGAAAGCAGCG	TTTCAACTGGTGGCTGCTAC
CaSUT1	TGGCAATTGTGTTCCCACAG	TGCCTCCACCAAATAGCTCA
CaSUT2	CGGTGCCTTGACTCTCTTTG	ACCTTGTCCTGAACCAGCAT
CaSUT3	TGAGCCTATGTGCAAGTGGA	AGGAACGCCAAGAAGTGAGA

Moreover, we explored the expression patterns of the *CaSUT* gene family in response to salt stress in leaves using the qRT-PCR analysis. Under high concentration NaCl (400 mM) treatment, the up-regulated expression of all *CaSUT* genes was very significant (*CaSUT1* and *CaSUT3*: >3-fold, *CaSUT2*: >16-fold) at 1 h at which the expression levels of all *CaSUTs* reached the highest (Figure 10 -c). However, only the expression levels of *CaSUT2* were upregulated at all the four time points. Transcript levels of *CaSUT1* showed a trend of upregulation and then downregulation. Down-regulation of *CaSUT3* was observed at 6 h and 12 h after salt treatment, but it exhibited only slight changes in expression level at 24 h compared with the control. According to our qRT-PCR data, *CaSUTs* have a certain function in response to abiotic stresses in pepper and their specific molecular mechanism might differ among genes.

## 4. Discussion

Sucrose transport from ‘source’ to ‘sink’ is one of the most important factors in plant growth and development. SUT (sucrose transporter) proteins, which are members of the MFS family, play a vital role in the partitioning of whole-plant carbohydrates [55,56]. In this study, putative *SUT* genes family of members in the published whole-genome sequences of the pepper (*Capsicum annuum*. L) and other 3 Solanaceae species (*S. lycopersicum, S. melongena* and *S. bicolor*) were analyzed. A total of 3 *SUT* genes in pepper were identified, which were similar to the grape (*Vitis vinifera, VvSUT1-3)*, but different from those in rice (*Oryza sativa L. OsSUT1-5*) [7] and Arabidopsis (*Arabidopsis thaliana, AtSUC1-9*) [49]. Interestingly, in tomato and potato, which also belong to Solanaceae as pepper, the same number of *SUT* family members was observed. This demonstrated that the number of *SUT* family members in Solanaceae was conserved, and the SUT gene family was small. It was well known that gene duplication (segmental and tandem) was common during the evolutionary process and was considered one of the fundamental driving forces of plant gene family expansion [57–60]. In our paper, most *SUTs* in Solanaceae were located on chromosomes 4 and 11, and we did not find specific-species genes duplication events in 4 Solanaceae species. Thus, we speculated that the *SUT* genes in each Solanaceae species might share similar duplication events during their long-term evolution process.

SUT proteins, known as sucrose/H^+^ cotransport proteins, are plant-specific and highly hydrophobic proteins with 12 transmembrane structural domains [61]. In this study, we found that most *SUTs* had 12 transmembrane structural domains, which was similar to the previous report [62]. In the present study, we also observed that the *CASUT2* and *SmSUT2* had 10 and 11 transmembrane structural domains, respectively, which were different from other SUT proteins. Further analysis found that the SUT proteins from other plant species (*Arabidopsis*, rice, grape and sorghum) that is close related with *CASUT2* and *SmSUT2* also had fewer transmembrane structural domains. We hypothesized that the absence of transmembrane helix domains of SUT proteins could be related to the structure variation of SUT proteins. At present, the specific mechanism needs to be explored in the future. In addition, we found that, compared with subgroup 1, subgroup 2 was closely related to subgroup 3. Subgroup 1 was composed of the *SUTs* from monocots and dicots, suggesting that the subgroup might have diverged from a single common ancestor prior to the mono-dicot split. We also found that the gene structure and conserved motifs among the three subgroups were different, implying that functions of *SUT* genes in Solanaceae species might have diverged during the evolution processes.

The promoter regions, which have many *cis*-elements, initiate the gene expression/transcription profiles in plant species. The promoter characteristic, such as the response to various stimuli, is determined by different combinations of regulatory *cis-*elements. Thus, investigation of regulatory *cis-*elements of target genes’ promoters can help us predict their expression patterns in response to diverse stimuli [58]. As one of the important economic crops, pepper faced variable environments during the whole growth and development process, while different environmental stimuli would affect their yield [63,64]. In this study, light and low-temperature responsive elements had been found in the promoters of the *CaSUT* gene family. Most members of the *CaSUT* genes contained one or two of the responsive elements above-mentioned, suggesting that the *SUT* genes family might be involved in the adaptation of environmental change in pepper. We also observed that *CaSUT* promoters contained many stress-related *cis-*elements, including MeJA, SA and ABA responsive elements, and the distribution of these stress-related *cis*-elements in pepper was similar to the previous report in oilseed rape [65]. MeJA, SA and ABA are primary phytohormones that are widely engaged in stress response in plants [66–68]. In this paper, MeJA responsive elements had been found in two of all the *CaSUT* genes (*CaSUT1* and *CaSUT2)*, and SA and ABA responsive elements were present in the promoter regions of *CaSUT1* and *CaSUT3*, indicating that the *CaSUT* genes could be associated with abiotic and biotic stress tolerance in pepper. In addition, we discovered that *CaSUT* promoters had gibberellin and auxin responsive elements, demonstrating that the *CaSUT* genes were involved in plant growth and development [69]. In short, in the present study, plenty of responsive *cis-*elements existed in *CaSUT* promoters, suggesting their key roles as crucial regulators in the response to abiotic and biotic stimuli, as well as their important function during pepper growth and development.

Spatial and temporal expression patterns of potential members of the gene family in plants contributed to understanding their function throughout their growth and development. Previous studies had reported that the *SUT* genes are expressed both in ‘source’ and ‘sink’ organs and can regulate the sucrose distribution and metabolism to satisfy the demands of plants during the growth and development process [70–72]. In this study, *CaSUT1* and *CaSUT2* showed high expression in all tested tissues, suggesting that they might be involved in pepper growth and development. Especially, the highest expression levels of *CaSUT2* were observed in leaves, we inferred that it might play a critical role in sucrose loading and unloading from ‘source’ to ‘sink’ organs [73,74]. In conclusion, the different expression patterns suggested that the *CaSUT* gene family could play roles in both ‘source’ and ‘sink’ organs. Moreover, our findings provided more information on the potential functionalities of the *SUT* genes in pepper.

Adverse environments affected seriously pepper growth and development, including cold, heat, and hormone stresses [73–78]. In the present study, *CaSUT1* and *CaSUT3* were up-regulated in roots under temperature treatments (cold and heat), suggesting that these genes were involved in roots in response to temperature stresses. Moreover, we observed that *CaSUT1* and *CaSUT3* had displayed similar expression patterns in roots under salt and hormone (SA and ABA) stresses (Figures 8 and 9), which indicated that both environmental stimulations could induce the expression of these genes. We also found that *CaSUT2* was relatively highly expressed in both leaves and roots under salt treatments, implying that its expression might be influenced by salt. Similar to salt treatment, *CaSUT2* was relatively highly expressed in all investigated organs, when pepper was exposed to cold, heat, and hormone stresses. *CaSUT2* was relatively highly expressed in all tested tissues in pepper, and responded to stress treatments very sensitively, which indicated that it might not only be involved in the loading and unloading of sucrose in phloem but also the response to environmental change. In short, the expression patterns of the *CaSUT* genes family under various stresses were different, indicating that members of the *CaSUT* gene family could have diverse functions to adapt to the altered environment.

Various abiotic stresses can affect the expression of the SUT gene, thereby influencing sucrose distribution in plants, and regulating osmotic balance and plant morphology [79,80]. Subsequently, the qRT-PCR was carried out to explore the expression patterns of the SUT gene family in pepper in response to cold, heat, and salt stresses. Interestingly, our qRT-PCR data demonstrated that *CaSUT* genes exhibited concurrent sensitivity to all abiotic stress treatments with uneven expression levels (Figure 10). Similar expression trends have been noticed in previous reports of the *SUT* gene family in rice [81,82], apple [83], and wheat [84]. In this paper, when exposed to heat stress, expression levels of *CaSUT1* were up-regulated at all stages, while *CaSUT3* increased at 12 h after treatment. Under salt stress, *CaSUT1* and *CaSUT3* were both obviously induced at 1 h. Under the above stresses, significantly up-regulated expression levels of *CaSUT2* were found in all stages analyzed, except for heat stress treatment. These findings suggested that *CaSUT* genes had different functions in growth and development, as well as in response to environmental stresses. However, more research into the specific regulatory mechanism is required.

## 5. Conclusion

In summary, the *SUT*s from 17 plant species were divided into 3 subgroups. The subgroup 1 had diverged from a single common ancestor prior to the mono-dicot split. Genome-wide characterization of the *SUT* genes in four Solanaceae species revealed that the SUT gene family in Solanaceae was highly conserved. Our results suggested that *CaSUT* could have diverse functions to adapt to adverse environments and advance our knowledge of the potential functions of SUT genes in Solanaceae species.
